# Hirano bodies differentially modulate cell death induced by tau and the amyloid precursor protein intracellular domain

**DOI:** 10.1186/1471-2202-15-74

**Published:** 2014-06-14

**Authors:** William Spears, Matthew Furgerson, John Michael Sweetnam, Parker Evans, Marla Gearing, Marcus Fechheimer, Ruth Furukawa

**Affiliations:** 1 Department of Cellular Biology, University of Georgia, Athens, GA 30602, USA; 2 Department of Biochemistry and Molecular Biology, University of Georgia, Athens, GA 30602, USA; 3 Pathology and Laboratory Medicine, Emory University School of Medicine, Atlanta, GA 30322, USA

**Keywords:** Hirano bodies, Actin, Tau, Amyloid precursor protein, Neurodegeneration, Alzheimer’s disease, Frontotemporal dementia

## Abstract

**Background:**

Hirano bodies are actin-rich paracrystalline inclusions found in brains of patients with Alzheimer’s disease (AD), frontotemporal dementia (FTD), and in normal aged individuals. Although studies of post-mortem brain tissue provide clues of etiology, the physiological function of Hirano bodies remains unknown. A cell culture model was utilized to study the interactions of mutant tau proteins, model Hirano bodies, and GSK3β in human astrocytoma cells.

**Results:**

Most tau variants showed co-localization with model Hirano bodies. Cosedimentation assays revealed this interaction may be direct, as recombinant purified forms of tau are all capable of binding F-actin. Model Hirano bodies had no effect or enhanced cell death induced by tau in the absence of amyloid precursor protein intracellular domain (AICD). In the presence of AICD and tau, synergistic cell death was observed in most cases, and model Hirano bodies decreased this synergistic cell death, except for forms of tau that caused significant cell death in the presence of Hirano bodies only. A role for the kinase GSK3β is suggested by the finding that a dominant negative form of GSK3β reduces this synergistic cell death. A subset of Hirano bodies in brain tissue of both Alzheimer’s disease and normal aged individuals was found to contain tau, with some Hirano bodies in Alzheimer’s disease brains containing hyperphosphorylated tau.

**Conclusion:**

The results demonstrate a complex interaction between tau and AICD involving activation of GSK3β in promoting cell death, and the ability of Hirano bodies to modulate this process.

## Background

The cause of sporadic Alzheimer’s disease (AD) is unknown, and an intricate interaction between multiple genetic, epigenetic, and environmental risk factors has been proposed (for review, see [1]). However, in approximately 1% of total AD cases, studies show that neurodegeneration results from mutations in the genes encoding the amyloid precursor protein (APP), presenilin 1 (PSEN1), or PSEN2 [2,3]. These mutations result in altered processing of APP, increased deposition of amyloid-beta (Aβ), and early onset neurodegeneration. This led to the formation of the amyloid cascade hypothesis, which posits that Aβ initiates a cascade of events leading to tau deposition, synaptic dysfunction and cognitive decline [4]. Intramembrane proteolysis of APP by γ-secretase also releases an intracellular fragment known as the amyloid precursor protein intracellular domain (AICD). AICD has been shown to form multi-protein complexes that regulate the induction of apoptosis (for review, see [5,6]). Multiple studies suggest that a downstream effector of AICD is the kinase GSK3β. Studies in neuronal cell cultures and a mouse model report that AICD induces upregulation of total levels of GSK3β as well as increased activation, and thus induces tau hyperphosphorylation [7-9]. Abnormal tau phosphorylation is an early marker of AD [10,11] and phosphorylated tau levels correlate with the severity of AD [12]. However, the precise mechanisms causing abnormal tau phosphorylation leading to pathological tau formation remain unclear.

Complementary to the amyloid cascade hypothesis was the discovery that mutations in tau cause a familial neurodegenerative disease called frontotemporal dementia with parkinsonism linked to chromosome 17 (FTDP-17) [13]. This disease and its sporadic (non-familial) counterparts, known as frontotemporal lobar degeneration with tau-positive inclusions (FTLD-tau), are characterized pathologically by the formation of neurofibrillary tangles (NFTs) and other neuronal and glial inclusions composed of hyperphosphorylated tau [14]. Aβ-containing plaques found in AD are not considered a significant invariant pathological feature of these tauopathies. Thus, the amyloid cascade hypothesis posits that Aβ acts upstream of tau to promote neurodegeneration. Nevertheless, significant plaque pathology is often reported in tauopathy patients’ brain tissue [15-19].

Hirano bodies are inclusion bodies found in significant numbers in the hippocampus in patients with AD compared to age matched controls [20]. In addition, Hirano bodies are found in postmortem brain tissue of patients afflicted with a variety of neurodegenerative and other diseases including the tauopathies, and in normal aged individuals [21,22], yet no studies to date explore the cellular basis of this finding. Although the contribution of Hirano bodies to tauopathy is unknown, cell culture and transgenic animal models of FTDP-17 suggest that alterations in the actin cytoskeleton and formation of Hirano bodies are an important event in the pathogenesis of tauopathies [23-25]. Tau has been previously shown to bind actin [26-28] and the interaction of tau with the actin cytoskeleton could play a role in disease progression. Hirano bodies are thought to be composed primarily of filamentous actin and actin-associated proteins [29-31], but they have also been shown to contain other components of the neuronal cytoskeleton such the microtubule associated protein tau [32,33]. Hirano bodies also accumulate a number of signaling proteins including the transcription factor FAC1 [34], transforming growth factor-β3 [35], and AICD [36,37]. Interestingly, Hirano et al., observed a positive correlation between the frequency of NFTs and Hirano bodies in patient samples [38]. In that study, Hirano bodies and NFTs were often found in the same cell, although the specific isoforms, mutants, or modifications on tau and physiological consequences of this are unknown.

Early studies of Hirano bodies were reliant on immunohistochemical staining of post-mortem brain tissue due to lack of a model system to study their formation or physiological function. We have developed a cell culture model of Hirano bodies by expressing a truncated form of 34-kDa protein (CT) from *Dictyostelium*[39-42]. Truncation of the amino terminus of 34-kDa protein to form CT results in a gain of function in activated actin binding activity and formation of model Hirano bodies in mammalian cells and in transgenic mice [41,43]. Model Hirano bodies mitigate the transcriptional activation activity of the APP intracellular domain (AICD), resulting in a decrease in cell death [37]. Further, model Hirano bodies decrease cell death potentiated by AICD and a pseudohyperphosphorylated (PHP) tau mimic [44].

We investigated the association of both wild type and mutant forms of tau with Hirano bodies, and their impact on cell death pathways involving tau and AICD. We report that nearly all tau isoforms and mutants tested associate with model Hirano bodies in cells, and bind to actin filaments *in vitro*. Cells expressing both tau and AICD exhibited cell death that was greater than the sum of that observed for tau and AICD alone. This synergistic cell death involved activation of GSK3β, and was eliminated by the presence of model Hirano bodies. These results extend previous reports affirming a specific role for Hirano bodies in aging and disease states and provides further evidence that the presence of Hirano bodies may have an important contribution to the pathogenesis of AD and tauopathies such as FTDP-17.

## Results

**Figure 1:**
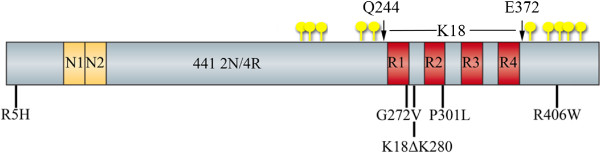
**Schematic illustration of tau isoforms, phosphorylation sites and mutations.** Point mutations R5H, G272V, P301L, and R406W were created in the 2N4R (441) tau isoform. Arrows represent boundaries of K18 (amino acids 244–372) [54]. N1 and N2 represent N-terminal exons 1 and 2. R1, R2, R3, and R4 represent microtubule binding domain repeats 1–4. Yellow circles designate 10 serine/threonine to glutamic acid mutations that occur in 352PHP tau [101]. This mutant was created in the 352 tau isoform lacking N1, N2, and R2.

The interaction of altered forms of tau with model Hirano bodies and the impact of this interaction on disease pathways leading to cell death was investigated in this study. Mutant tau proteins were chosen on the basis of previous characterization of differential biochemical properties and their effects on cell death. Expression of mutant tau found in FTDP-17 (R5H, G272V, P301L, R406W) has differential effects on cell death when expressed in cell cultures and in various animal models of tauopathy [24,45,46] and these effects may be attributable to the biochemical properties of tau [47,48]. The tau mutant ΔK280 is representative of a group of FTDP-17 mutants previously shown to have increased susceptibility to aggregation compared to WT tau [47-49]. However, whether or not tau polymerization is directly neurotoxic continues to be a major question (for review, see [1]). Previous studies have established a cell culture, *C. elegans*, and mouse model of tau aggregation through expression of a tau fragment comprising only the microtubule binding domain with deletion of lysine 280 (K18ΔK280), which has a high propensity for β-structure and aggregation [50-53]. Studies show that expression of K18ΔK280 tau is more lethal than the same fragment without the deletion of lysine 280 (K18), and causes cell death prior to the formation of mature aggregations [50,54,55]. The nomenclature of the tau variants and position of the mutation(s) used in this study are shown in Figure 1.

### FTDP-17 tau mutants and Hirano bodies differentially modulate cell death

**Figure 2:**
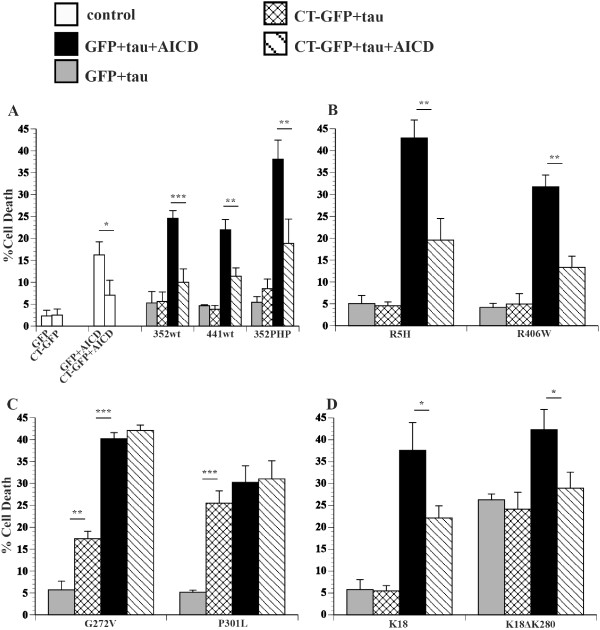
**FTDP-17 tau and model Hirano bodies differentially modulate cell death.** H4 cells were transiently transfected with equal amounts of plasmid DNA encoding AICD and/or a single tau variant in the presence or absence of model Hirano bodies (CT-GFP). Model Hirano bodies and a tau variant in the absence of AICD (check bars) or presence of AICD (striped bars). GFP and a tau variant in the absence of AICD (gray bars) or the presence of AICD (black bars). **A**. Tau variants 352WT, 441WT, or 352PHP. **B**. Tau variants R5H or R406W. **C**. Tau variants G272V and P301L. **D**. Tau variants K18 and K18ΔK280. Model Hirano bodies protect from cell death induced by AICD and tau, except for tau mutants G272V and P301L. Note that G272V and P301L potentiate cell death in the presence of model Hirano bodies alone. *p < 0.05, **p < 0.01, ***p < 0.001. Error bars represent the standard deviation.

The effect of FTDP-17 tau mutants (R5H, G272V, P301L, R406W), 352PHP, and truncated K18 and K18ΔK280 tau on cell death in the presence of model Hirano bodies was investigated. As expected, exogenous expression of wild type tau, 352PHP, and FTDP-17 tau mutants alone, R5H, R406W, G272V, or P301L did not produce significant cell death (Figure 2). Consistent with previous reports, expression of K18ΔK280 resulted in significant cell death compared to expression of K18 (Figure 2D, gray bars ***p < 0.001). Coexpression of either 352WT, 441WT, 352PHP, R5H, R406W, K18, or K18ΔK280 tau in the presence of model Hirano bodies resulted only in background levels of cell death (Figure 2AB, check bars). In contrast, expression of either G272V (**p < 0.01) or P301L (***p < 0.001) in the presence of model Hirano bodies resulted in significant potentiation of cell death (Figure 2C, check bars).

### Actin binding

**Figure 3:**
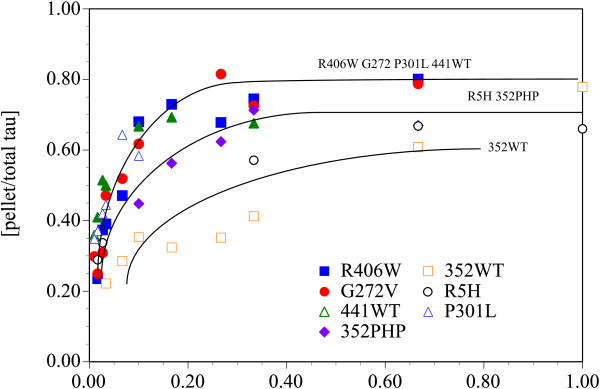
**Relative binding of recombinant tau to F-actin.** Tau binds differentially to F-actin with binding of R406W (blue square), G272V (red circle), P301L (blue triangle), and 441WT (green triangle) > 352PHP (purple diamond) and R5H (black circle) > 352WT (orange square). The curves are to aid the reader and do not indicate biochemical binding curves. The standard deviations were not shown for clarity.

Tau has been previously shown to bind F-actin – albeit not to saturation – and form F-actin bundles in solution [26-28]. We determined the relative F-actin binding for wild type tau and mutant forms of tau to investigate whether the strength of actin binding may correlate with cell death induced by tau and Hirano bodies. Using recombinant protein purified from *E.coli* and cosedimentation with mixtures of F-actin, wild type and mutant forms of tau do not achieve saturation binding to F-actin as shown in Figure 3. R406W, G272V, P301L, and 441WT bind F-actin better than 352PHP and R5H, which are greater than 352WT (Figure 3). This result is consistent with previous results of wild type recombinant tau [27].

We investigated whether tau has an effect on formation of model Hirano bodies since a prior report found that tau promotes the formation of Hirano bodies [56]. Transient expression of CT-GFP to induce model Hirano bodies and either 352WT, 441WT, 352PHP, or P301L for 48 h did not cause a change in the size of model Hirano bodies (Additional file 1). Thus, Hirano bodies can form in the absence of tau, and the presence of various forms of tau does not modulate the formation of Hirano bodies.

### Hirano bodies, tau, and AICD

Since previous reports have indicated that model Hirano bodies protected against AICD-induced cell death in the presence of 352WT or 352PHP [44], we investigated whether model Hirano bodies would have an effect on cell death induced by FTDP-17 tau (R5H, G272V, P301L, R406W), or truncated tau (K18 or K18ΔK280) in the presence of AICD. Expression of AICD resulted in modest levels of cell death (Figure 2A, white bars), and the presence of model Hirano bodies significantly lowered this death (white bars, *p < 0.05). Coexpression of either 352WT/AICD or 441WT/AICD (black bars) caused an incremental increase in cell death that is similar to what is expected from the additive effects of AICD alone and wild type tau alone (see Table 1). In contrast, a marked potentiation in cell death was observed upon coexpression of 352PHP/AICD (black bars, Table 1) consistent with previous data [44]. The potentiation of cell death is a synergistic interaction between AICD and 352PHP since the predicted amount of cell death due to AICD alone plus that obtained with 352PHP alone is significantly less than observed when the two are present together (Table 1). The presence of model Hirano bodies protected against cell death induced by either 352WT/AICD (stripe bar, ***p < 0.001) or 441WT/AICD (stripe bar, **p < 0.01), or 352PHP/AICD (stripe bar, ** p < 0.01).

**Table 1:** Additive versus synergistic cell death induced by co-expression of AICD and tau

**Tau**	**% Cell death (Additive) = GFP/AICD + GFP/tau**	**% Cell death ± SD (Actual) = GFP/AICD/tau**	**p value**
352tauWT	22.3% ± 1.46	24.6% ± 1.74	N.S.
441tauWT	20.9% ± 2.78	22.0% ± 2.36	N.S.
**352tauPHP**	**21.7% ± 1.98**	**38.1% ± 4.35**	**p < 0.05**
**R5H**	**21.3% ± 2.49**	**42.9% ± 4.09**	**p < 0.01**
**K18**	**22.0% ± 4.24**	**39.0% ± 6.98**	**p < 0.05**
K18ΔK280	42.5% ± 4.28	42.3% ± 4.61	N.S.
**P301L**	**21.4% ± 2.97**	**30.2% ± 3.79**	**p < 0.05**
**G272V**	**21.9% ± 2.73**	**40.2% ± 1.40**	**p < 0.01**
**R406W**	**20.4% ± 3.93**	**31.7% ± 2.70**	**p < 0.05**

Values of cell death obtained when GFP/AICD or GFP/tau transiently expressed were added together and compared to the actual values of cell death obtained when GFP/AICD/tau were co-expressed. Synergistic cell death (bold entries) was observed when AICD was co-expressed with 352PHP, R5H, K18, P301L, G272V, or R406W. N.S. = not significant.

The effect of FTDP-17 tau mutants on AICD-induced cell death in the presence or absence of model Hirano bodies was investigated. Similar to 352PHP tau, FTDP-17 tau mutants (R5H, G272V, P301L, R406W) enhance cell death in synergy with AICD (Figure 2B,C, black bars). The amount of cell death observed is greater than obtained from AICD alone plus that of tau alone (Table 1). The presence of model Hirano bodies protected from cell death due to either AICD/R5H or AICD/R406W (Figure 2B, stripe bars **p < 0.01). However, in cells expressing either G272V/AICD or P301L/AICD, model Hirano bodies had no effect on cell death (Figure 2C, stripe bars). Expression of K18 and K18ΔK280 with AICD increased cell death synergistically and additively, respectively (black bars, *p < 0.05; Table 1). The presence of model Hirano bodies lowered cell death (stripe bars *p < 0.05) for both K18 and K18ΔK280. These data suggest that model Hirano bodies have a differential effect on cell death induced by tau and/or AICD, depending on the tau variant used.

Collectively, the synergistic versus additive cell death of co-expression of AICD and tau variants suggests a connection by one or more molecular pathways. The effect of Hirano bodies on cell death may affect AICD, or tau, and/or the synergy that arises from their interaction. Possible routes of this interaction could involve activation of the protein kinase GSK3β, phosphorylation of tau, binding of tau to F-actin, or differential aggregation kinetics of the tau variants.

### Hirano bodies differentially influence cell death induced by GSK3β and tau

**Figure 4:**
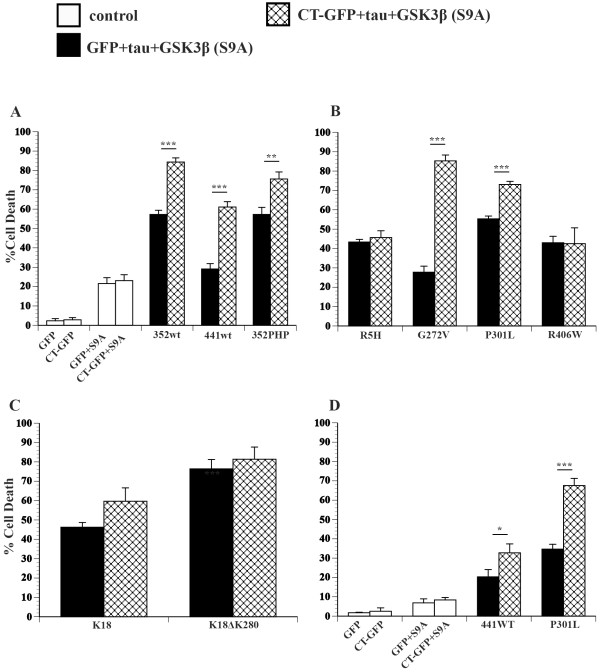
**Model Hirano bodies differentially influence cell death in the presence of GSK3β (S9A) and tau.** H4 cells were transiently transfected with equal amounts of plasmid DNA. Control cells (white bars) were transfected with plasmid encoding either GFP or model Hirano bodies (CT-GFP) in the presence or absence of constitutively active GSK3β (S9A). Cells were transfected with plasmid encoding GSK3β (S9A), a single tau variant, and either GFP (black bars) or model Hirano bodies (check bars). **A**. Tau variants 352WT, 441WT, or 352PHP. **B**. Tau variants R5H, G272V, P301l, or R406W.** C**. Tau variants K18 or K18ΔK280.** D**. Tau variants 441WT or P301L transfected with a lower amount of plasmid encoding GSK3β (S9A). Model Hirano bodies significantly promote cell death induced by GSK3β (S9A) and tau except in tau mutants R5H, R406W, K18, and K18ΔK280, where they have no effect. *p < 0.05, **p < 0.01, ***p < 0.001. Error bars represent the standard deviation.

GSK3β has been implicated in the pathogenesis of AD (for review, see [57]). Specifically, GSK3β is thought to become upregulated and/or activated by Aβ, AICD, and the c-terminal 31 amino acids of APP (c31), triggering a cascade of signaling events leading to tau hyperphosphorylation, apoptosis, and cell death [7,9,58]. The contribution of GSK3β and tau to cell death in the presence of model Hirano bodies was investigated using a constitutively active and a dominant negative form of GSK3β. Exogenous expression of a constitutively active mutant of GSK3β (S9A) alone causes cell death similar to that of AICD, and the presence of model Hirano bodies has no effect on this death (Figure 4A, white bars). In contrast, exogenous expression of GSK3β (S9A) in the presence of 352WT or 352PHP causes significant potentiation of cell death compared to GSK3β (S9A) alone (Figure 4A, black bars ***p < 0.001 or **p < 0.01, respectively, Table 2). Interestingly, although 352WT and 352PHP produce similar amounts of cell death under these conditions, 441WT expression causes only an incremental increase in cell death when expressed with GSK3β (S9A), which is not significantly different from that expected from cell death due to 441WT alone + GSK3β (S9A) alone (Figure 4A, black bar; Table 2). Exogenous expression of GSK3β (S9A) with either WT tau isoform or 352PHP in the presence of model Hirano bodies causes even further potentiation of cell death (Figure 4A, check bars, ***p < 0.001 or **p < 0.01).

**Table 2:** Additive versus synergistic cell death induced by co-expression of tau and GSK3β (S9A)

**Tau**	**% Cell death (Additive) = GFP/GSK3β(S9A) + GFP/tau**	**% Cell death (Actual) = GFP/GSK3β(S9A)/ tau**	**p value**
**352tauWT**	**24.1% ± 1.44**	**57.3% ± 1.74**	**p < 0.005**
441tauWT	24.7% ± 0.95	29.1% ± 2.77	N.S.
**352tauPHP**	**25.5% ± 1.52**	**49.8% ± 3.63**	**p < 0.005**
**R5H**	**25.1% ± 1.62**	**43.4% ± 1.37**	**p < 0.005**
**K18**	**25.9% ± 1.40**	**46.3% ± 2.44**	**p < 0.005**
**K18ΔK280**	**46.4% ± 1.00**	**76.4% ± 4.84**	**p < 0.005**
**P301L**	**25.2% ± 1.37**	**55.3% ± 1.45**	**p < 0.005**
G272V	25.8% ± 2.88	27.8% ± 3.13	N.S.
**R406W**	**24.3% ± 1.14**	**43.0% ± 3.33**	**p < 0.005**

Values of cell death obtained when GFP/GSK3β (S9A) or GFP/tau transiently expressed were added together and compared to the actual values of cell death obtained when GFP/GSK3β (S9A)/tau were co-expressed. Synergistic cell death (bold entries) was observed when GSK3β (S9A) was co-expressed with 352PHP, R5H, K18, K18ΔK280, P301L, or R406W. Interestingly, cell death in the presence of G272V was not affected by the presence of GSK3β (S9A). N.S. = not significant.

The effect of FTDP-17 mutant tau expression on cell death induced by GSK3β (S9A) and model Hirano bodies was investigated. Exogenous expression of tau mutants R5H, P301L, and R406W in the presence of GSK3β (S9A) increased cell death synergistically compared to the sum of that observed with expression of GSK3β (S9A) and tau alone (Figure 4B, black bars; Table 2). However, expression of G272V tau in the presence of GSK3β (S9A) resulted in only additive increases in cell death compared to expression of GSK3β (S9A) alone (Figure 4AB, Table 2). We also observed differences in the ability of model Hirano bodies to modulate cell death in the presence of FTDP-17 mutant tau and GSK3β (S9A). Model Hirano bodies enhanced cell death in the presence of GSK3β (S9A)/G272V tau and GSK3β (S9A)/P301L tau (Figure 4B, check bars ***p < 0.001), a result that occurred when either G272V or P301L was expressed with model Hirano bodies. However, model Hirano bodies had no effect on cell death induced by GSK3β (S9A)/R5H tau or GSK3β (S9A)/R406W tau (Figure 4B, check bars).

GSK3β (S9A) also significantly enhanced cell death in the presence of K18 or K18ΔK280 compared to the sum of the expression of GSK3β (S9A) and tau alone (Figure 4C, black bars ***p < 0.001; Table 2). Model Hirano bodies did not have a statistically significant effect on this cell death (Figure 4C, check bars). We also measured cell death under conditions in which expression of GSK3β (S9A) did not induce significantly greater levels of cell death than GFP controls (Figure 4D), showing that GSK3β (S9A)-induced cell death is dose dependent. Similar to previous results (Figure 4A-C), exogenous expression of GSK3β (S9A)/441WT or GSK3β (S9A)/P301L potentiated cell death compared to GSK3β (S9A) alone (Figure 4D, black bars). In addition, the presence of model Hirano bodies further increased this cell death compared to expression of GSK3β (S9A)/441WT (check bars, *p < 0.05) or GSK3β (S9A)/P301L (check bars ***p < 0.001). These results show that model Hirano bodies are not protective against cell death induced by GSK3β and tau, and depending on the mutation, may further increase cell death.

The contribution of dominant negative GSK3β (K85A) [7,59] was used to test the role of GSK3β in cell death induced by tau and model Hirano bodies. GSK3β (K85A) was transiently co-expressed with model Hirano bodies in the presence and absence of G272V (Table 3). The unexpectedly higher level of cell death due the presence of G272V and model Hirano bodies was reduced in the presence of GSK3β (K85A) to that expected from the control GFP/G272V + GFP/GSK3β(K85A) (Table 3, p < 0.02). This result shows an interaction between F-actin and tau that is more complex than F-actin binding alone and that phosphorylation of tau likely plays a role in cell death.

**Table 3:** The effect of tau phosphorylation on cell death in the presence of model Hirano bodies

**Sample**	**%Cell death**
Hirano body/GSK3β(K85A)	4.20 ± 0.77
Hirano body/G272V	17.90 ± 2.59
Hirano body/G272V/GSK3β(K85A)	10.20 ± 1.35
GFP/G272V	4.75 ± 0.74
GFP/G272V/ GSK3β(K85A)	5.80 ± 0.56
GFP/G272V + GFP/ GSK3β(K85A)	8.95 ± 2.06

Expression of GSK3β(K85A) reduced the synergistic cell death observed when both G272V and model Hirano bodies are present, to an additive effect, implying that the phosphorylation state is important.

**Figure 5:**
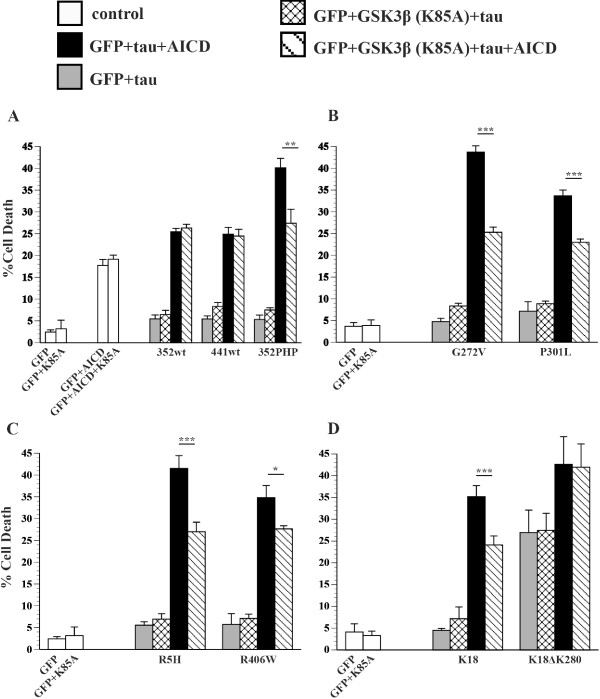
**GSK3β (K85A) differentially influences cell death in the presence of AICD and tau.** H4 cells were transiently transfected with equal amounts of plasmid DNA encoding GFP and different single tau variants in the absence of AICD (grey bars) or presence of AICD (black bars) or dominant negative GSK3β (K85A) and tau in the absence of AICD (check bar) or presence of AICD (striped bar). **A**. Tau variant 352WT, 441WT, or 352PHP. **B**. Tau variant G272V or P301L. **C**. Tau variant R5H or R406W. **D**. Tau variant K18 or K18ΔK280. GSK3β (K85A) significantly decreases cell death due to AICD and tau except in tau variants 352WT, 441WT, K18ΔK280 where there is no effect. *p < 0.05, **p < 0.01, ***p < 0.001. Error bars represent the standard deviation.

To determine whether cell death induced by combinations of AICD and tau involve activation of GSK3β and the phosphorylation state of tau, the effect of a dominant negative GSK3β (K85A) was investigated (Figure 5). Expression of GSK3β (K85A) with GFP does not significantly increase the cell death due to GFP alone (Figure 4, white bars). Further, expression of AICD induces death of approximately 17% of cells, and this number is not significantly changed in the presence of GSK3β (K85A) (Figure 5A, white bars). Thus, cell death due to AICD alone does not require activation by GSK3β. Cell death due to wild type, 352PHP, FTDP-17 mutants, and K18 and K18ΔK280 was unaffected by expression of GSK3β (K85A) (Figure 5A and D, grey and checked bars). Cell death due to tau + AICD was suppressed by expression of GSK3β (K85A) for 352PHP, G272V, P301L, R5H, and R406W, and K18 (Figure 5A-D, black and striped bars). However, expression of GSK3β (K85A) had no effect on cell death induced by AICD/352WT, AICD/441WT, or AICD/K18ΔK280 (Figure 5A and D, black and striped bars). Interestingly, cell death of all tau forms + AICD that were suppressed by expression of GSK3β (K85A) showed synergistic cell death in the presence of GSK3β (S9A) (Table 2). These results imply that synergistic cell death due to AICD and tau involve activation of GSK3β, and that phosphorylation of tau is an important factor to cell death. Cell death due to tau forms + AICD that were not suppressed by expression of GSK3β (K85A) had levels of cell death that was not significantly different from the sum of that observed with tau and AICD separately.

### Phosphorylation of tau

**Figure 6:**
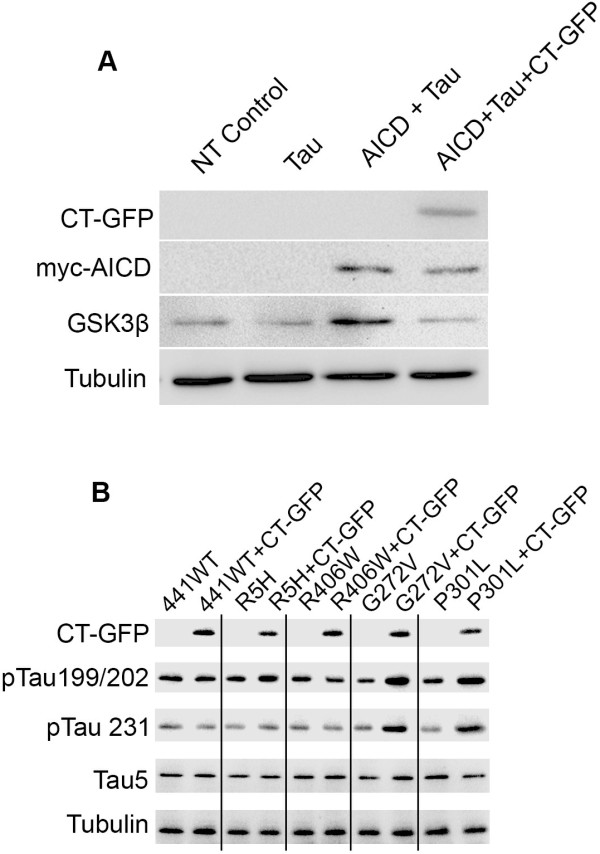
**Western blot analysis of GSK3β levels and tau phosphorylation.** HEK293 cells were transiently transfected with equal amounts of plasmid. **A**. Expression of exogenous AICD increases total GSK3β protein. Expression of CT-GFP in addition to AICD reduces GSK3β to control levels. Expression of tau has no effect on levels of GSK3β. NT = Not Transfected control. **B**. The presence of exogenous CT-GFP increases phosphorylation of G272V and P301L at Ser199/Ser202 and Thr231. Expression of CT-GFP has no effect on 441WT, R406W, or R5H tau phosphorylation at any epitope probed.

Results of cell viability in the presence of GSK3β (K85A) suggest that 352PHP, R5H, R406W, G272V, and P301L become phosphorylated by GSK3β in the presence of AICD or CT-GFP. Previously, others have reported that AICD is capable of increasing cellular levels of GSK3β [7-9]. Therefore, western blot analysis was performed to determine whether the presence of AICD and/or CT-GFP impact the amount of GSK3β protein in our cell culture system. The presence of exogenous AICD increases total GSK3β levels above that of untransfected cells and also of cells expressing tau only (Figure 6A). Furthermore, expression of CT-GFP in the presence of AICD and tau reduces the amount of GSK3β to that of the control (Figure 6A). The increase of tau-induced cell death due to the presence of G272V or P301L and CT-GFP was reduced in the presence of GSK3β K85A (Figure 5), suggesting CT-GFP may promote phosphorylation of these tau mutants. To test this hypothesis, western blot analysis was performed on different tau mutants to determine if the presence of CT-GFP increases phosphorylation at specific amino acids. 441WT, R406W, and R5H tau showed no increase in phosphorylation at Ser199, Ser202, or Thr231 in the presence of CT-GFP (Figure 6B). Interestingly, both P301L and G272V have increased phosphorylation at Ser199 and Ser202 and Thr231 in the presence of CT-GFP (Figure 6B). P301L and G272V exhibited synergistic cell death in the presence of CT-GFP (Figure 2C). These results show that the ability of specific mutant tau proteins to induce cell death in the presence of AICD or CT-GFP is due to GSK3β phosphorylation of tau.

### Localization of tau and model Hirano bodies

**Figure 7:**
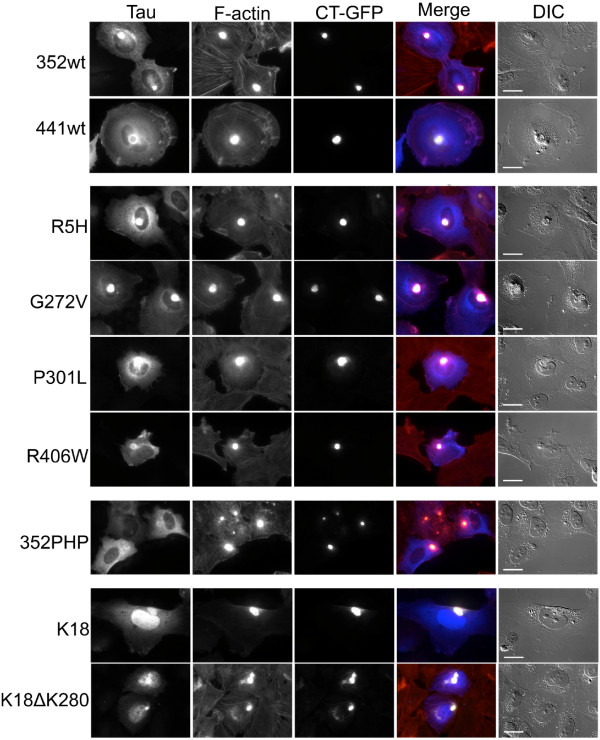
**Immunofluorescence localization of tau and model Hirano bodies.** H4 cells were transiently transfected with FLAG-tagged tau constructs and CT-GFP to induce model Hirano bodies. 24 h post-transfection, cells were fixed and stained for good preservation of F-actin. Tau was stained with anti-FLAG primary antibody and Alexa Fluor350 secondary antibody (blue). F-actin was stained using TRITC-phalloidin (red). All tau isoforms colocalize with model Hirano bodies except 352PHP, which is localized diffusely throughout the cell. Tau variants examined were control tau 352WT, 441WT, or tau mutants K18, R5H, G272V, P301L, R406W, K18ΔK280, and 352PHP. Scale bar = 20 μm.

Since preferential accumulation of different forms of tau might indicate physiological function of Hirano bodies, mutant tau and model Hirano body localization was investigated. H4 cells were transiently transfected with CT-GFP to induce model Hirano bodies in the absence or presence of one of the following FLAG-tagged tau constructs: 441WT, 352WT, R5H, G272V, P301L, R406W, 352PHP, K18ΔK280 and K18. All FTDP-17 mutant tau proteins as well as WT tau isoforms were present throughout the model Hirano body or formed a ring-like structure around the periphery of the model Hirano body shown in Figure 7. The exception was 352PHP, which was diffusely localized throughout the cell. Further, truncated tau mutants (K18 or K18ΔK280) also showed a strong nuclear localization. These results show that regardless of mutations, tau shows enrichment at sites of F-actin accumulation. However, hyperphosphorylation may decrease this association, as shown for 352PHP tau.

**Figure 8:**
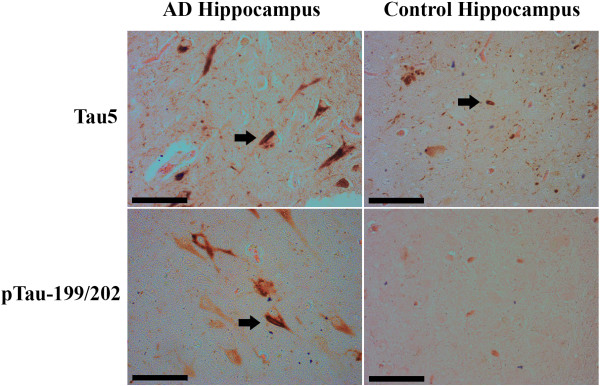
**Immunohistochemistry localization of tau and Hirano bodies in human brains.** Immunohistochemistry was performed on paraffin embedded human brain sections using Tau5 or pTau-199/202 antibodies following an eosin counterstain to visualize Hirano bodies (arrows). Tau5 antibodies colocalized with Hirano bodies in both Alzheimer diseased and control brains while pTau-199/202 colocalized with Hirano bodies only in Alzheimer diseased brains. Scale bar = 50 μm

Localization of tau in human brain tissue was performed to confirm that tau is present in Hirano bodies. In AD and control brain sections, 4.1 and 2.7% of Hirano bodies are well stained for tau, respectively (Figure 8). Localization of phosphorylated tau (pTau199/202) to Hirano bodies was observed only in AD brain sections and comprised 2.9% of the total Hirano bodies. These findings are in agreement with prior studies that report tau localized in a fraction of all Hirano bodies in human hippocampus [32,33].

## Discussion

Tau contributes significantly to neurodegeneration in FTDP-17, Alzheimer’s disease and other tauopathies by aggregating to form oligomers, paired helical filaments (PHF), and eventually large aggregates including neurofibrillary tangles (NFTs). *In vitro*, formation of oligomers and higher order polymers of tau is thought to occur through the process of nucleation, elongation, and/or autocatalytic growth [60,61]. Regulatory pathways may influence the availability of free tau, its phosphorylation state, propensity to aggregate, and inducers of aggregation. Dissecting the formation of oligomeric and higher order polymers of tau *in vivo* and their contribution to cell death require probing these pathways. In this study we have investigated the role of Hirano bodies in modulating tau localization and cell death due to tau.

### Proposed cell death pathway involving Hirano bodies, tau, AICD, and GSK3β

**Figure 9:**
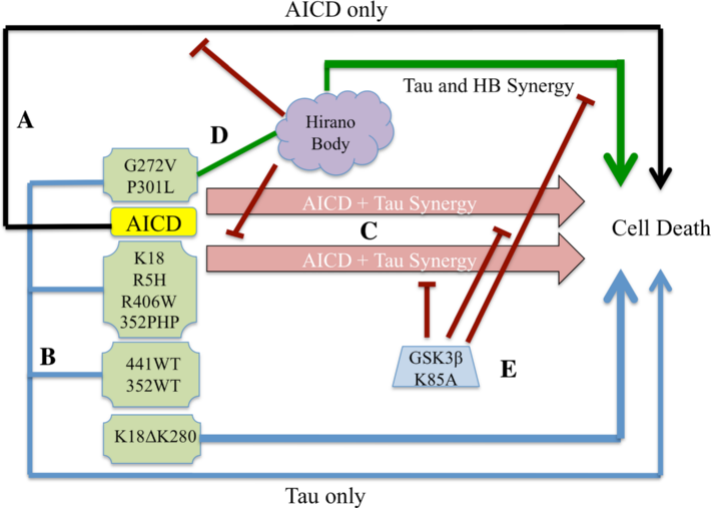
**Model of the interplay between AICD, tau, and model Hirano bodies. A**. The black line represents cell death initiated by “AICD only” pathway involving Fe65, Tip60, p53, and caspases (41, 6). **B**. Blue line represent the modest levels of cell death initiated by “tau only” including K18ΔK280 alone and low levels of cell death from all other tau alone (green boxes). **C**. The pink arrows represent the synergistic cell death due to AICD and either G272V, P301L, K18, RH5, R406W, or 352PHP tau. 441WT, 352WT, and K18ΔK280 do not synergize with AICD to promote cell death**. D**. Model Hirano bodies protect against AICD -induced cell death and cell death initiated by AICD and K18, RH5, R406W, or 352PHP tau (red lines). In contrast, model Hirano bodies potentiate AICD- induced cell death in the presence of either P301L or G272V tau (green lines). Model Hirano bodies alone have no impact on K18, R5H, R406W, 352PHP, 441WT, 352WT, or K18ΔK280 tau -induced cell death or have negative consequences. In addition, the synergistic cell death induced by AICD in the presence of G272V or P301L tau is not affected by the presence of model Hirano bodies. **E**. Expression of the dominant negative GSK3-β (K85A) prevents cell death initiated by synergistic interaction of AICD and tau or model Hirano bodies and G272V or P301L tau (red lines).

The role of tau in cell death induced by the presence or absence of model Hirano bodies and/or AICD is a complex intersection of several variables: 1) affinity of tau for actin; 2) the phosphorylation state and location of the phosphorylated amino acids on tau; and 3) the kinetics and propensity of tau to self-aggregate that could depend on phosphorylation or other modification. As shown in Figure 9, AICD causes significant cell death when expressed alone. Similarly, expression of K18ΔK280 causes significant cell death unlike expression of all other forms of tau tested (Figure 9). When coexpressed with AICD, all tau mutants expressed except for 441WT, 352WT, and K18ΔK280 produced synergistic cell death (Figure 9C). This cell death was ameliorated in the presence of model Hirano bodies, except in the case of P301L or G272V tau (red lines). This could be due to reduction of AICD or tau through indirect or direct binding to F-actin in the model Hirano bodies and/or reduction of GSK3β (Figure 6A) and thus affecting the phosphorylation state of tau (Figure 6B). Interestingly, the expression of model Hirano bodies and P301L or G272V tau led to significant cell death in the absence of AICD (Figure 2C). This process could be facilitated by a combination of binding to F-actin, increased phosphorylation that reduces F-actin binding, and subsequent tau aggregation. The affinity of tau for F-actin itself is insufficient as R406W and 441WT bound F-actin as well as G272V and P301L.

We also find that expression of a dominant negative form of GSK3β mitigates cell death due to the synergistic effect of AICD and tau in most cases (Figure 5, Figure 9E). This result underscores the importance of GSK3β activation and tau phosphorylation in the synergistic interaction of AICD and tau. In support of this, expression of dominant negative GSK3β (K85A) does not reduce cell death due to expression of AICD alone, or AICD and K18ΔK280, as most GSK3β phosphorylation sites are absent from this mutant. We show that expression of GSK3β (K85A) ameliorates cell death due to G272V in the presence of Hirano bodies, further substantiating the role of GSK3β phosphorylation in our system, even in the absence of exogenously expressed AICD. In summary, these results support the assertion that a combination of tau phosphorylation and aggregation leads to cell death, which may be either enhanced or mitigated by the presence of model Hirano bodies depending on the form of tau and state of its modification.

These new results resolve an apparent contradiction in previous literature. Expression of mutant forms of tau were shown to induce cell death only after overexpression of actin and subsequent formation of actin-rich structures in a *Drosophila* model [24]. However, model Hirano bodies were previously reported to reduce toxicity associated with tau under certain conditions [44]. Here we show that Hirano bodies can both promote and protect against cell death initiated by tau depending on the type of tau expressed.

### Tau and actin

Multiple studies point to the role of tau as an actin binding protein [25-28,62-66]. However, this idea is not without controversy. Studies of tau-actin interactions utilize a range of experimental approaches that yield differential results [67]. Tau has been shown to bind and bundle actin *in vitro*[24,27,28,62]. Tau bound weakly to actin and did not reach saturation [27]. In competitive binding studies with actin, the preferred substrate of tau is tubulin polymerized into microtubules [27,65]. Our study is the first to show that all tau mutants tested are able to bind to F-actin with the resultant binding R406W, G272V, P301L, and 441WT > 352PHP and R5H > 352WT (Figure 3). R406W, G272V, and P301L have the highest affinity for F-actin yet only G272V and P301L potentiate cell death in the presence of model Hirano bodies and/or AICD (Figure 2, Table 1). The differences in F-actin binding affinity of the various tau mutants does not explain the differential effect of tau on cell death in the presence of model Hirano bodies. However, since recombinant protein produced in *E.coli* was used, the impact of tau post-translational modifications on F-actin binding was not measured. Other results reported in this study imply that the phosphorylation state of tau is important. In the presence of constitutively active GSK3β (S9A), model Hirano bodies potentiate cell death in the presence of tau (see Table 2). In addition, only a small percentage of Hirano bodies in AD brain contain phosphorylated tau (Figure 8). Another group was unable to detect pTau-202/205 in Hirano bodies from Alzheimer’s patients [68]. It is possible that colocalization of tau with Hirano bodies varies from patient to patient and is heavily influenced by post-translational modification, and aggregation state given that Hirano bodies frequently occur in NFT-containing neurons [38]. Consistent with this hypothesis, others have shown that phosphorylation of tau at certain sites reduces, but may not abolish its ability to associate with actin filaments [69]. Conversely, studies have suggested that minor phosphorylation of certain residues may enhance actin binding [24,27,70]. This suggests a complex, dynamic relationship between tau and actin. This may explain why 352PHP binds F-actin *in vitro* (Figure 3), but does not co-localize with model Hirano bodies (Figure 7). *In vivo*, tau association with actin has been shown to be critical for tau-induced neurodegeneration [24]. We cannot rule out that other modes of actin and tau interaction may be occurring such as intermediate binding proteins or other protein modifications. For example, tau also binds to Fe65 [71], and Fe65 is known to be enriched in Hirano bodies [37].

### Phosphorylation of tau

Studies characterizing FTDP-17 tau mutants demonstrated that tau was highly phosphorylated, and depending on the mutation, differentially phosphorylated [72-74]. This differential phosphorylation also affects the ability of tau and its mutants to bind to microtubules [75], affects microtubule dynamic instability, and promotes microtubule assembly (for a review, see [76]). It is also well known that tau phosphorylation at multiple sites *in vivo* decreases its association with microtubules [67,77], thus making tau available to aggregate. Hyperphosphorylated tau has a tighter, more folded conformation and in some cases, an increased propensity to aggregate [14,74,78-83].

In all tau constructs tested except for R5H, R406W, K18, or K18ΔK280, expression of exogenous constitutively active GSK3β (S9A) and tau in the presence of model Hirano bodies strongly potentiated cell death compared to expression of GSK3β (S9A) and tau (Figure 4). Since GSK3β phosphorylates many substrates, results need to be interpreted with some caution. However, our results suggest that GSK3β or another kinase induces formation of hyperphosphorylated tau species. Model Hirano bodies could potentiate cell death by increasing the local concentration of tau through F-actin binding, serving as the nucleation agent in subsequent tau phosphorylation and aggregation. G272V and P301L have a higher propensity to aggregate than 441WT (see below [47]), and may therefore aggregate and induce cell death more easily in the presence of Hirano bodies. G272V and P301L were more phosphorylated in the presence of model Hirano bodies (Figure 6), lending credence to this supposition. Further experiments show that expression of dominant negative GSK3β (K85A) in the presence AICD and tau decreases levels of cell death to that expected from an additive effect of cell death induced by AICD + tau alone (Figure 2, Table 1). However, model Hirano bodies did not further enhance cell death in the presence of R406W shown previously to be hypophosphorylated in the presence of GSK3β [7,58] or tau mutants that did not contain the majority of GSK3β phosphorylation sites (K18 and K18ΔK280). Expression of dominant negative GSK3β (K85A) in the presence of G272V and model Hirano bodies lowered cell death to that expected of an additive effect of cell death induced by G272V + model Hirano bodies alone (Figure 5). This may suggest a complementary role for phosphorylation and aggregation.

Consistent with this, *in vitro* studies characterizing the 352PHP tau mutant show that these mutations collectively inhibit aggregation compared to WT tau [84]. Therefore, transient phosphorylation and dephosphorylation of tau may be critical to formation of a pathologically relevant tau species. This may explain why expression of 352PHP tau and model Hirano bodies does not cause increased cell death in our system. It has been shown that phosphorylation of specific residues on tau is required to prime tau for additional phosphorylation [85-88], and that 352PHP becomes phosphorylated in cell cultures under certain conditions at other serine/threonine residues that are not mutated [89]. Therefore, the amino acid charge substitutions used to create the phosphorylation mimics on 352PHP may prime phosphorylation at other sites. This could explain why expression of either AICD or GSK3β with 352PHP results in a synergistic cell death phenotype. These results support the hypothesis that model Hirano bodies enhance cell death in the presence of highly phosphorylated tau, and reduce cell death in the presence of other tau variants.

### Aggregation of FTDP-17 mutant tau

The ability of tau to promote cell death in the presence of model Hirano bodies and/or AICD correlates most strongly with the propensity of tau to self-aggregate. Multiple studies show that FTDP-17 tau mutants vary widely in their ability to form PHFs and NFTs *in vitro*, in cell culture, and *in vivo* as well as the kinetics in which they form [90]. Studies with recombinant tau obtained from bacteria showed that ΔK280(441tau) had a greater propensity to form paired helical filaments than P301L > G272V > R406W > 441WT with a faster kinetic speed [47]. Tau mutants P301L and K18ΔK280 show the greatest tendency to aggregate [47], and potentiated the highest levels of cell death in the presence of model Hirano bodies. All tau mutants except for K18ΔK280 were incapable of inducing cell death alone consistent with previous reports [50,54]. The ability of K18ΔK280 to cause cell death was attributed to the ability of this fragment to aggregate since mutation of hexapeptide motifs in tau essential for β-structure and aggregation reduced cell death and rescued neurodegeneration [55,91]. Model Hirano bodies have no effect on cell death initiated by expression of exogenous K18ΔK280. Because this mutant is capable of initiating cell death on its own, it likely does not need model Hirano bodies to further aid its aggregation. Although little is known about the R5H tau mutation, R5L has been characterized *in vitro* with increased nucleation rate of aggregation and increased numbers of tau filaments [92], a trait shared with R5H [18]. R406W tau does not robustly aggregate *in vitro* or in cell cultures [92-95]. In fact, R406W tau has been shown to be hypophosphorylated in resting cells compared to WT tau [93,95], and hypophosphorylated when exposed to GSK3β *in vitro*[94]. Thus, tau phosphorylation may be related to tau aggregation and cell death. The specific tau species responsible for potentiating cell death in our model is unknown, and more studies are needed to determine whether further tau modification and proteolytic processing contributes to cell death. In support of a relationship between aggregation and cell death, some FTDP-17 patients exhibit more aggressive symptoms than AD patients, coincident with robust tau pathology at an earlier age of onset (for review, see [96]).

A possible mechanism for the interaction of model Hirano bodies and tau is that model Hirano bodies increase the local concentration of tau through a direct or indirect interaction. All the full-length tau proteins bound F-actin in our study and all tau proteins with the exception of 352PHP were enriched with model Hirano bodies (Figures 3 and 7). While tau binds with a lower affinity to actin than microtubules, the concentration of F-actin in a Hirano body could be 360–600 μM based on the actin concentration in F-actin solutions bundled by the *Dictyostelium* 34 kDa actin bundling protein [97]. The high local concentration of F-actin in a model Hirano body could compensate for weak tau binding. Phosphorylation, aggregation, or other protein modifications could become more favorable due to the high local concentration. Tau mutants most prone to aggregation then initiate cell death. Previous studies with recombinant tau induced to aggregate by the presence of heparin showed that both P301L and G272V aggregated faster and to a greater extent than R406W or 441WT [47]. The truncated K18ΔK280 aggregated exceptionally quickly in contrast to K18. Model Hirano bodies were not able to protect against cell death in the presence of these aggregation-prone tau mutants alone or in addition to AICD. In contrast, WT tau isoforms and tau mutants R406W and R5H did not cause cell death in the presence of model Hirano bodies, and model Hirano bodies were able to protect from cell death induced by these tau mutants in the presence of AICD. These forms of tau were slower to aggregate and did so to a smaller extent than P301L, G272V, or K18ΔK280 [47].

### AICD and tau

Our data is consistent with the idea that tau significantly contributes to cell death in the presence of AICD. This complements previous results showing that expression of exogenous c31/APP/352PHP tau or AICD/352PHP tau results in a potentiation of cell death compared to cells expressing AICD or 352PHP alone [44]. In this study, expression of all tau forms tested enhanced cell death in the presence of AICD (albeit at different levels) to a level that was significantly greater than the sum of AICD and tau expressed separately with the exception of 352WT, 441WT, and K18ΔK280 (Table 1). It is noteworthy that exogenous expression of dominant negative GSK3β (K85A) with AICD and all forms of tau that previously induced synergistic cell death decreased to an additive sum of AICD alone plus tau alone, while AICD and all forms of tau that do not induce synergistic cell death were unaffected by GSK3β (K85A). In addition, the presence of model Hirano bodies with AICD and tau decreased the amount of GSK3β. These results support a model in which AICD potentiates cell death in the presence of tau and that phosphorylation of tau is important to that process. AICD has been shown to up-regulate or be upstream of GSK3β activation [7]. Our results strongly support the conclusion that the interplay between AICD and tau involves activation of GSK3β and phosphorylation of tau, and that there are at least two major effects of AICD. AICD alone activates Fe65, Tip60, and caspases, while the interaction of AICD with tau to cause cell death requires activation of GSK3β.

## Conclusion

We provide further evidence that Hirano bodies have a specific effect on the pathogenesis of neurodegenerative disease, with respect to tau and C-terminal fragments of APP. We suggest that Hirano bodies do not serve a general protective function by simply accumulating cytosolic proteins, but rather protect against cell death initiated by AICD and mitigate or enhance cell death dependent on the biochemical properties of tau. This data complements key discoveries made in transgenic animal models and neuronal cell culture reporting abnormal rearrangement of F-actin during cell stress or neurodegeneration, and suggest that Hirano bodies play a complex role in the pathogenesis of disease.

## Methods

### Plasmids

CT-EGFP was utilized to induce model Hirano bodies as previously described [42,44]. Other plasmids utilized encoded GFP (pEGFP-N1, Clontech, Mountain View, CA), AICD (APPc58-myc, c-terminal 58 amino acids of APP-695, a generous gift from Bradley Hyman, Harvard Medical School) [98], and HA-tagged GSK3β (Addgene plasmid 15994) [99]. GSK3β (S9A) (constitutively active) [100], and GSK3β (K85A) (dominant negative) [7,59] were constructed from HA-GSK3β using a QuikChange II XL Site-Directed Mutagenesis Kit (Stratagene, La Jolla CA) using mutagenic primers K85A 5’GAACTGGTCGCCATCAAGAAAGTATTGCAGGAC 3’ and S9A 5’ CCAGAACCACCGCCTTTGCGGAGAGC 3’. The coding sequences of these plasmids were confirmed by sequencing.

A single MAPT gene in the central nervous system generates six tau isoforms by alternative splicing of its pre-mRNA. Isoforms ranging in size from 352–441 residues differ by exclusion or inclusion of exons 2,3, and 10. Plasmids were constructed to express wild type tau (352WT or 441WT) or mutant forms (352PHP, R5H, G272V, P301L, R406W, K18, or K18ΔK280) in either mammalian or bacterial cells. 352PHP is a tau mutant created in the shortest tau isoform (352WT) in which 10 serine/threonine residues (S198, S199, S202, T231, S235, S396, S404, S409, S413, and S422) were mutated to glutamic acid to mimic a hyperphosphorylated state (generous gifts from Roland Brandt, University of Osnabrück, Osnabrück, Germany) [101] as shown in Figure 1. K18 comprises the microtubule-binding domain (amino acids Q244 to E372, numbering from the 441 length tau) [47]. To generate N-terminal FLAG-tagged tau constructs with a CMV promoter for expression in mammalian cells (Figure 1), the coding sequence of 352PHP was digested from the 352PHP plasmid with ClaI. Using the coding sequence of 441 length human WT tau (441WT) in pET-29b (Addgene plasmid 16316) [102] as a template, ClaI restriction sites were introduced through PCR, and 441WT coding sequence was cloned into the ClaI site of the remainder of the 352PHP plasmid backbone containing the FLAG-tag and CMV promoter. Tau mutants in the 441 length tau in either the mammalian or bacterial expression plasmids were constructed using a QuikChange II XL Site-Directed Mutagenesis Kit (Stratagene, La Jolla CA). Primers for mutagenesis were: ΔK280 (deletion of K280) 5’ GCAGATAATTAATAAGCTGGATCTTAGC, R5H 5’ATGGCTGAGCCCCACCAGGAGTTCGAAG, G272V 5’GCACCAGCCGGGAGTCGGGAAGGTGCAG, P301L 5’ATCAACACGTCCTGGGAGGCGGCAG, and R406W 5’GGACACGTCTCCATGGCATCTCAGCAATG. Tau constructs K18 and K18ΔK280 were generated by the introduction of ClaI sites to the 5’ and 3’ end of K18 or K18ΔK280 using either the 441WT or ΔK280 441WT as template, digesting the PCR products, and ligating into the ClaI site of the remainder of the 352PHP plasmid backbone after deletion of the coding sequence. The coding sequences of all plasmids were verified by sequencing.

### Immunofluorescence

Hirano bodies have been proposed to have a glial origin [103], and because glial cells have been shown to be important mediators of cell death induced by Aβ and tau [104], H4 human astroglioma cells were utilized. Furthermore, H4 cells have been shown to contain nearly undetectable levels of endogenous tau [105,106]. H4 astroglioma cells (American Type Culture Collection, Manassas, VA) were cultured in Dulbecco’s Modified Eagle Medium (DMEM) supplemented with 10% fetal bovine serum (Atlanta Biologicals, Flowery Branch, GA) at 37°C and 5% CO_2_. H4 cells were plated onto glass coverslips and allowed to adhere for 24 h. Cells were transiently transfected with equal amounts of plasmid (1 μg each) using Lipofectamine LTX (Invitrogen, Carlsbad, CA) according to manufacturer’s instructions. Cells were processed after 24 h for immunofluorescence as previously described [42]. Coverslips were visualized with a Zeiss Axioobserver Z1 equipped with an AxioCam MRm controlled by AxioVision4.6 software. Antibodies used were anti-FLAG rabbit antibody to label tau (Sigma-Aldrich, St. Louis, MO), and Alexa Fluor 350 conjugated anti-rabbit secondary antibody (Molecular Probes, Eugene, OR). F-actin was visualized using TRITC-conjugated phalloidin (Sigma-Aldrich, St. Louis, MO), and nuclei were stained with DAPI (Sigma-Aldrich, St. Louis, MO). Characterization of the types of actin-rich structures was performed on H4 cells transfected as described above and processed for immunofluorescence 48 h after transfection. CT-GFP-rich structures were characterized having normal cellular localization, fibrillar structures, Hirano bodies or large Hirano bodies. Hirano body size was determined using NIH ImageJ using the threshold function to eliminate user bias. Large Hirano bodies are those in the fourth quartile. Significance between samples was determined using the non-parametric Mann–Whitney test. Experiments were performed in a minimum of triplicate.

### Immunohistochemistry

All samples were de-identified and coded only by sample number (see Table 4), and supplied as eight-micron, paraformaldehyde-fixed paraffin sections mounted on slides. Mounted sections were dewaxed in xylene and rehydrated in an ethanol gradient prior to antigen retrieval in boiling 10 mM sodium citrate plus 0.05% Tween 20 pH 6.0 for 20 minutes. Endogenous peroxidase activity was inhibited by incubating sections in 3% hydrogen peroxide for 10 minutes prior to washing with phosphate buffered saline (PBS) and blocking with 10 mg/ml bovine serum albumin in PBS overnight. Samples were stained for total tau using mouse tau 5 antibody (1/400 in PBS, 4% BSA) (Santa Cruz Biotechnology, Dallas, TX), or tau phosphorylated at serine 199/202 using rabbit anti-pTau199/202 (1/300 in PBS, 4% BSA) (Sigma-Aldrich Chemical Co, St. Louis, MO). Anti-mouse and anti-rabbit biotinylated secondary antibodies (1/800 in PBS) (Sigma-Aldrich Chemical Co, St. Louis, MO) followed by a streptavidin-HRP polymer complex (1/1000 in PBS) with diaminobenzidine enhanced substrate system (Vector Laboratory, Burlingame, CA) were utilized as a detection system. Samples were counterstained with hematoxylin/eosin (Sigma-Aldrich Chemical Co, St. Louis, MO) to visualize Hirano bodies. All protocols were approved by the University of Georgia Institutional Biosafety Committee and the Institutional Review Board.

**Table 4:** Samples of human hippocampus were obtained from the Alzheimer’s Disease Research Center at Emory University

**Case number**	**Age at death**	**Race/sex**
AD-1	81	Male
AD-2	83	Caucasian male
AD-3	66	African American female
Control-1	88	Caucasian female
Control-2	94	Caucasian male

Samples were screened for presence of Hirano bodies, and were derived from patients with AD, or control subjects that had no known history of neurological disease and no neuropathological changes indicative of neurodegenerative disease at autopsy.

### Cell death assays

24 hours prior to transfection, 8,000 H4 cells/well were plated into 96-well plates (Nalge Nunc, Rochester, NY). Cells were transiently transfected with equal amounts of plasmid (250 ng each) using Lipofectamine LTX (Invitrogen, Carlsbad, CA) according to manufacturer’s instructions. In some experiments, the amount of GSK3β (S9A) plasmid was reduced (150 ng) to examine its dose dependent effect while the amounts of the other plasmids were kept constant. 24 hours post-transfection, cell death assays were performed as previously described [44]. Briefly, cells were incubated with 9 nM Sytox Orange, a cell impermeable nucleic acid dye (Invitrogen, Carlsbad, CA), and 264 μM Hoechst 33258 (Sigma-Aldrich Chemical Co, St. Louis, MO) for 15 min at 37°C and 5% CO_2_. Due to inefficient transfection, GFP was used as a transfection control. Sytox Orange positive fluorescence was used to indicate dead cells. A minimum of 80 cells was counted per sample and each condition was sampled at least three independent times. Analysis of statistical significance was performed using Student’s t-test. The interactions of multiple components and their contributions to cell death were analyzed by using propagation of error, comparing the calculated sum and standard deviations of cell death observed when the two components were separate to that observed when they were present in the assay together to determine the error of the calculated sum. This strategy and applying Student’s t-test allowed determination of whether the cell death observed when the two components were present together was significantly greater than expected, suggesting a biological and/or molecular interaction.

### Protein purification

Expression of wild type and mutated forms of tau in pET29-b was induced by the addition of 1 mM IPTG in *E.coli* Bl-21(DE-3) with the exception of 352PHP which required no IPTG. Recombinant tau proteins were purified as previously described [107] with the following modifications. Cells were lysed by the addition of 0.2 mg/ml of lysozyme in the bacterial resuspension buffer and stirred until lysis was complete. The lysate was sonicated prior to the addition of NaCl and subsequent boiling. The lysate was clarified by ultracentrifugation and the supernatant was diluted with cation exchange buffer A without NaCl to a final concentration of 50 mM NaCl, and applied to CM-cellulose column (GE Healthcare Life Sciences, Piscataway, NJ). The column was washed with cation exchange buffer A until the OD reached baseline levels and subsequently eluted with a linear gradient 0.05–0.4 M NaCl gradient. Fractions containing tau were identified by SDS-PAGE, collected, diluted with cation exchange buffer A without NaCl to a final concentration of 50 mM NaCl and concentrated on CM-cellulose. The mutant tau was eluted with cation exchange buffer with 0.6 M NaCl. Fractions containing the mutant tau were identified by OD280 nm, sedimented at 13,000 × g for 15 min at 4°C, and the supernatant applied directly to sephacryl S-200 (GE Healthcare Life Sciences, Piscataway, NJ) column equilibrated with 10 mM HEPES, pH 7.4, 150 mM NaCl, 1 mM DTT, and 0.02% sodium azide. Fractions containing tau were identified by SDS-PAGE. Individual fractions containing the highest concentrations of tau were frozen at -80°C and used without further concentrating. Five hours prior to use, the tau was dialyzed versus cosedimentation buffer (see below).

Actin was prepared from rabbit skeletal muscle acetone powder [108,109] and further purified by application to sephadex G-150. The actin was maintained for up to 1 week in cosedimentation buffer (10 mM PIPES, pH 7.0, 100 mM KCl, 1 mM MgCl_2_, 1 mM ATP, 1 mM DTT, and 0.02% sodium azide) with daily buffer changes. The protein concentrations of actin and tau were determined using bicinchoninic acid method (Pierce Protein Biology, Rockford, IL) [110] with bovine serum albumin as a standard.

Cosedimentation assays were performed as previously described [111]. F-actin and/or tau mixtures were incubated 2 h prior to ultracentrifugation. The samples were analyzed by SDS-PAGE and quantified utilizing ChemiDoc™ MP System and Image Lab™ software (Bio-Rad Laboratories, Hercules, CA). The amount of tau in the pellets was corrected for the small amount of tau alone found in the pellet. Experiments were performed in a minimum of triplicate.

### Western blot

HEK293T cells (American Type Culture Collection, Manassas, VA) were cultured in Dulbecco’s Modified Eagle Medium (DMEM) supplemented with 10% fetal bovine serum (Atlanta Biologicals, Flowery Branch, GA) at 37°C and 5% CO_2_. They were plated at a density of 4 × 10^5^ cells per 60 mm diameter dish. Cells were transfected with equal amounts of plasmid (2.5 μg each) using Lipofectamine 2000 (Invitrogen, Carlsbad, CA) according to manufacturer’s instructions. At 48 hours after transfection, cells were washed twice with ice-cold PBS and lysed with 20 mM Tris, pH 7.5, 1% triton-X100, 0.1% SDS, 0.5% deoxycholic acid, and 10% glycerol with 10 μL protease inhibitor cocktail (5 mM EGTA, 1 mM DTT, 100 mM leupeptin, 10 mM pepstatin, 0.1 M PMSF, 0.1 M benzamidine, and 0.5 M ϵ-aminocaproic acid). Cell debris was separated from total homogenate by centrifugation at 13,000 g for 15 min at 4°C. The supernatant was stored at −80°C until used. Protein concentrations of the supernatants were determined by bicinchoninic acid assay using BSA as a standard [110]. For western blot analysis, samples were loaded at equal total protein, separated by SDS-PAGE, and transferred to nitrocellulose membranes. Blots were blocked in 5% nonfat dry milk in TBST and probed using with either rabbit anti-pTau199/202 (1/4500) (Sigma-Aldrich Chemical Co, St. Louis, MO), rabbit-anti pTau231 (1/2000) (Acris Antibodies, San Diego, CA), goat-anti pTau396 (1/2000) (Santa Cruz Biotechnology, Dallas, TX), rabbit anti-GFP (1/5000) (Sigma-Aldrich Chemical Co., St. Louis, MO), mouse anti-tau5 (1/5000) (Santa Cruz Biotechnology, Dallas, TX), mouse anti-alpha tubulin (1/8000) (Millipore, Billerica, MA), mouse anti-myc (1/8000) (Cell Signaling Technology, Danvers, MA), or rabbit anti-GSK-3β (1/1000) (Santa Cruz Biotechnology, Dallas, TX). After three washes with TBST, blots were incubated with either goat anti-mouse horseradish peroxidase, goat anti-rabbit horseradish peroxidase (1/10000) (Pierce-lab, Rockford, IL), or donkey anti-goat horseradish peroxidase (1/8000) (Millipore, Billerica, MA) and detected by chemiluminescence using SuperSignal Western Dura Extended Duration Substrate (Thermo Scientific, Rockford IL). Images were captured utilizing ChemiDoc™ MP system and Image Lab™ software (Bio-Rad Laboratories, Hercules, CA).

Blots were performed in duplicate.

## Competing interests

The authors declare that they have no competing interests.

## Authors’ contributions

All authors in this manuscript contributed to the design of the study, analysis/ interpretation of data, and drafting of this manuscript. WS, MFurgerson, JMS, PE, and RF carried out all experiments under the supervision of MFechheimer and RF. WS and MFurgerson performed the cell death assays and mutagenesis. WS and JMS performed the immunofluorescence staining. MFurgerson and PE performed the immunohistochemistry staining. RF performed the protein purification and F-actin cosedimentation assay. MG sectioned and supplied human brain tissue samples. All authors have read and approved this manuscript for publication.

## Supplementary Material

Additional file 1: Figure S1 Mutant tau does not affect model Hirano body formation. H4 cells were transiently transfected with equal amounts of plasmid DNA encoding CT-GFP to induce model Hirano bodies in the absence (white bars) or presence of either 352WT (stripe bar), 441WT (grey bar), 352 PHP (black bar), or P301L (crosshatch bar). Cells were fixed after 48 hrs. Model Hirano bodies were characterized as normal, fibrillar, Hirano body, or large Hirano body as determined by GFP fluorescence. There is no difference between the populations of cells. Scale bar = 20 μm. Click here for file
