# Supplementary figures and images for: Hirano bodies differentially modulate cell death induced by tau and the amyloid precursor protein intracellular domain
Source: BMC Neurosci. 2014 Jun 14;15:74. doi: 10.1186/1471-2202-15-74 (PMC4084581; doi:10.1186/1471-2202-15-74)

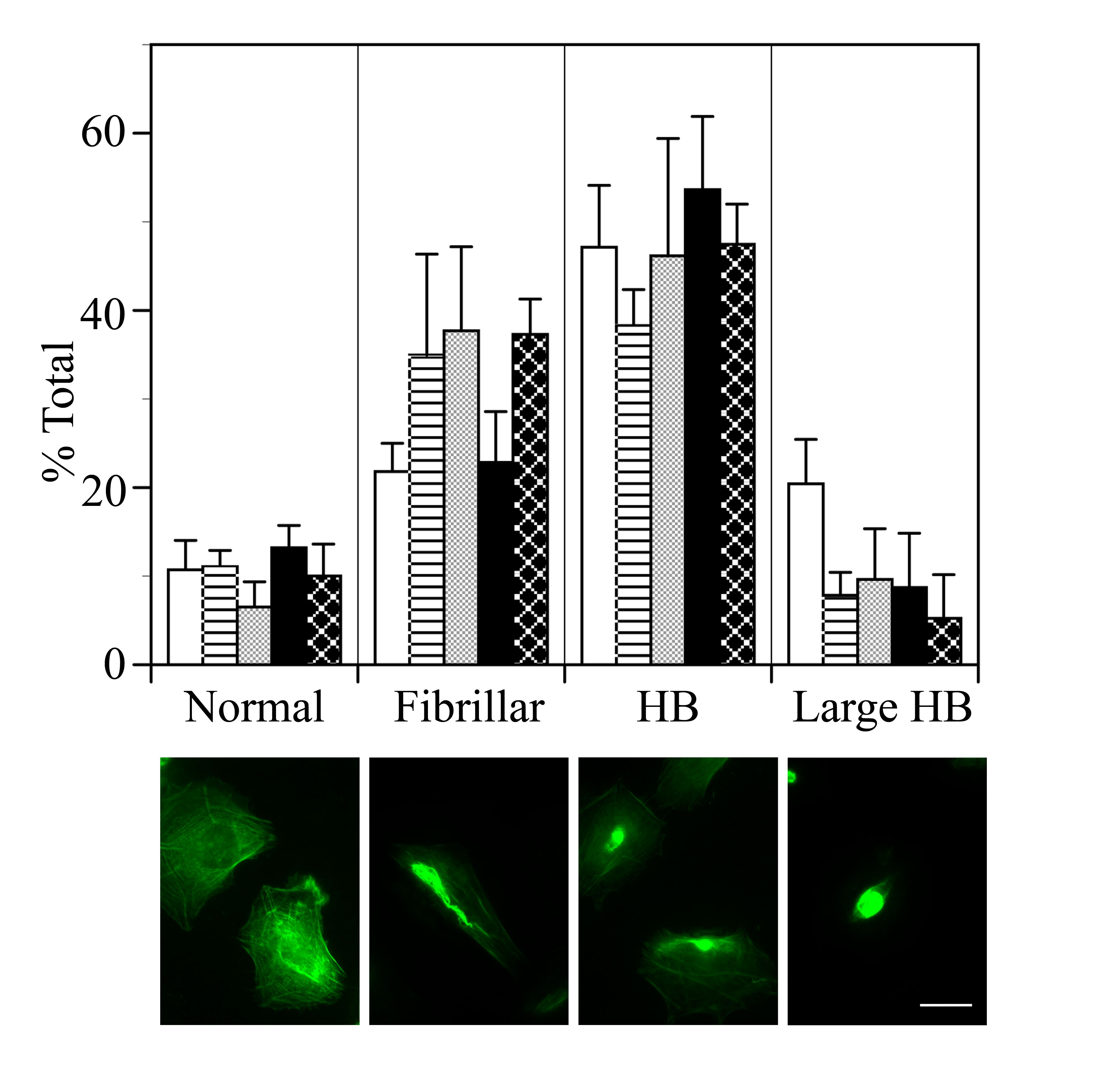

Supplement: Additional file 1: Figure S1 — Mutant tau does not affect model Hirano body formation. H4 cells were transiently transfected with equal amounts of plasmid DNA encoding CT-GFP to induce model Hirano bodies in the absence (white bars) or presence of either 352WT (stripe bar), 441WT (grey bar), 352 PHP (black bar), or P301L (crosshatch bar). Cells were fixed after 48 hrs. Model Hirano bodies were characterized as normal, fibrillar, Hirano body, or large Hirano body as determined by GFP fluorescence. There is no difference between the populations of cells. Scale bar = 20 μm. [file 1471-2202-15-74-S1.png]
